# Testing the effects of mass drug administration of azithromycin on mortality and other outcomes among 1–11-month-old infants in Mali (LAKANA): study protocol for a cluster-randomized, placebo-controlled, double-blinded, parallel-group, three-arm clinical trial

**DOI:** 10.1186/s13063-022-06966-7

**Published:** 2023-01-03

**Authors:** Laura Adubra, Dagmar Alber, Per Ashorn, Ulla Ashorn, Yin Bun Cheung, Elaine Cloutman-Green, Fatoumata Diallo, Camilla Ducker, Riku Elovainio, Yue-Mei Fan, Lily Gates, Gwydion Gruffudd, Tiia Haapaniemi, Fadima Haidara, Lotta Hallamaa, Rikhard Ihamuotila, Nigel Klein, Juho Luoma, Owen Martell, Samba Sow, Taru Vehmasto, LAKANA Trial Team

**Affiliations:** 1 Center for Child, Adolescent and Maternal Health Research, Faculty of Medicine and Health Technology, Tampere University, Tampere, Finland; 2 Great Ormond Street Institute of Child Health, University College London, London, UK; 3 Department of Paediatrics, Tampere University Hospital, Tampere, Finland; 4 Program in Health Services and Systems Research and Centre for Quantitative Medicine, Duke-NUS Medical School, Singapore, Singapore; 5 Center for Vaccine Development, Bamako, Mali; 6 Tro Da Ltd, London, UK

**Keywords:** Randomized controlled trial, Infant, Antibiotic, Azithromycin, Placebo, Mortality, Morbidity, Growth, Infection, Inflammation, Antimicrobial resistance

## Abstract

**Background:**

Mass drug administration (MDA) of azithromycin (AZI) has been shown to reduce under-5 mortality in some but not all sub-Saharan African settings. A large-scale cluster-randomized trial conducted in Malawi, Niger, and Tanzania suggested that the effect differs by country, may be stronger in infants, and may be concentrated within the first 3 months after treatment. Another study found no effect when azithromycin was given concomitantly with seasonal malaria chemoprevention (SMC). Given the observed heterogeneity and possible effect modification by other co-interventions, further trials are needed to determine the efficacy in additional settings and to determine the most effective treatment regimen.

**Methods:**

LAKANA stands for Large-scale Assessment of the Key health-promoting Activities of two New mass drug administration regimens with Azithromycin. The LAKANA trial is designed to address the mortality and health impacts of 4 or 2 annual rounds of azithromycin MDA delivered to 1–11-month-old (29–364 days) infants, in a high-mortality and malaria holoendemic Malian setting where there is a national SMC program. Participating villages (clusters) are randomly allocated in a ratio of 3:2:4 to three groups: *placebo* (control):*4-dose AZI*:*2-dose AZI*. The primary outcome measured is mortality. Antimicrobial resistance (AMR) will be monitored closely before, during, and after the intervention and both among those receiving and those not receiving MDA with the study drugs. Other outcomes, from a subset of villages, comprise efficacy outcomes related to morbidity, growth and nutritional status, outcomes related to the mechanism of azithromycin activity through measures of malaria parasitemia and inflammation, safety outcomes (AMR, adverse and serious adverse events), and outcomes related to the implementation of the intervention documenting feasibility, acceptability, and economic aspects. The enrolment commenced in October 2020 and is planned to be completed by the end of 2022. The expected date of study completion is December 2024.

**Discussion:**

If LAKANA provides evidence in support of a positive mortality benefit resulting from azithromycin MDA, it will significantly contribute to the options for successfully promoting child survival in Mali, and elsewhere in sub-Saharan Africa.

**Trial registration:**

ClinicalTrials.gov NCT04424511. Registered on 11 June 2020.

**Supplementary Information:**

The online version contains supplementary material available at 10.1186/s13063-022-06966-7.

## Introduction

In 2020, an estimated 5 million children died before reaching their fifth birthday [1]. Sub-Saharan Africa (SSA) persistently experienced the highest under-5 mortality rate (U5MR) at 74 deaths per 1000 live births, 14 times higher than the risk for children in Europe and North America [1]. Despite sustained efforts by the global health community, and investments in key interventions such as routine immunizations, promotion and support of breastfeeding, nutrient supplementation, and access to safe water, sanitation, and hygiene (WASH), the SSA region remains off-track for reaching the Sustainable Development Goal target of 25 or fewer deaths under the age of 5 years per 1000 live births by 2030 [2]. The leading causes of death in this age group include pre-term birth complications, birth asphyxia/trauma, and infectious diseases, such as lower respiratory tract infections, diarrhea, and malaria [3].

Mass drug administration (MDA) of azithromycin, a broad-spectrum macrolide antibiotic, has been identified as a potential high-impact intervention to promote child survival in countries with high under-5 mortality largely driven by infectious causes [4]. Azithromycin MDA has been used for decades in the context of trachoma control programs [5], and in some settings, prevention of other infections such as malaria, diarrhea, and pneumonia has been reported [6–11]. Reduction in childhood mortality has also been reported in randomized controlled trials on trachoma [12–14]. These findings encouraged further investigations, notably the Macrolides Oraux pour Réduire les Décès avec un Oeil sur la Résistance (MORDOR) study [4]. MORDOR was a large-scale randomized placebo-controlled trial in Malawi, Niger, and Tanzania, which demonstrated a statistically significant 13.5% all-cause mortality reduction in 1–59-month-old children receiving biannual azithromycin MDA. The point estimates for mortality differences were largest in certain subgroups including among the youngest children, and at the Niger site, a country where the childhood mortality rate is extremely high. In another large trial in Mali and Burkina Faso, azithromycin MDA when given to 3–59-month-old children over three consecutive months with seasonal malaria chemoprevention (SMC) did not reduce a combined outcome of hospitalization and deaths, compared to SMC alone [15]. In 2020, based on the available evidence, the World Health Organization (WHO) issued a conditional guideline on the use of azithromycin MDA for child survival [16]. The guideline specified targeting high-mortality settings, i.e., where infant mortality rate (IMR) is > 60 per 1000 live births or U5MR is > 80 per 1000 live births, and 1–11-month-old infants. The rationale for the narrow age range is to target the subgroup for which the greatest benefit was suggested, and to alleviate the risk of emergence of antimicrobial resistance (AMR) [17, 18]. The WHO guideline highlighted the need for further research including on optimal dose, frequency and number of intervention cycles, and potential harms, notably AMR.

As part of a larger effort to build the evidence base for the potential impact of azithromycin on childhood mortality, we are conducting in Mali a randomized placebo-controlled trial evaluating the impact on mortality of 4 or 2 annual rounds of azithromycin MDA delivered to 1–11-month-old infants. In 2020, Mali was reporting the world’s seventh highest national U5MR, estimated at 91 deaths per 1000 live births [1]. In a subset of villages selected for AMR detection and monitoring, we will also investigate the impacts on other health outcomes, and the mechanism of action of azithromycin through measures of markers of infection, and inflammation. The trial name, LAKANA, stands for Large-scale Assessment of the Key health-promoting Activities of two New mass drug administration regimens with Azithromycin. In Bambara, the language spoken in the study area, LAKANA means “to protect” or “to be safe.”

## Methods

### Study design

**Fig. 1:**
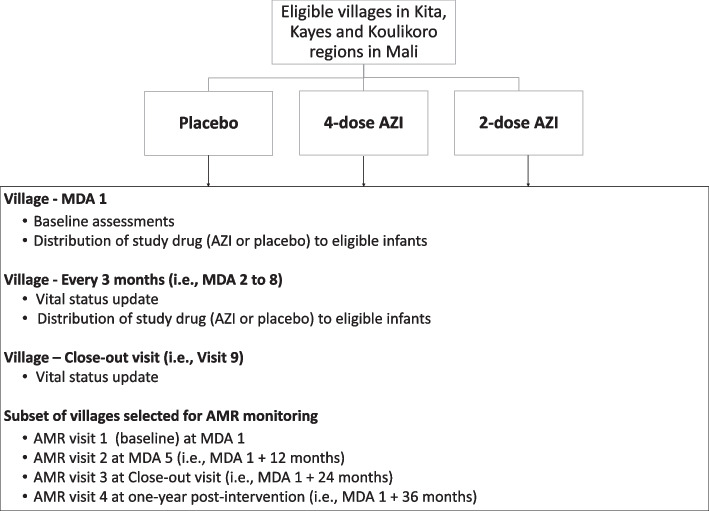
LAKANA trial design summary. Eligible infants in the villages randomized to (i) placebo (control), receive placebo mixture every 3 months; (ii) 4-dose AZI, receive azithromycin every 3 months; (iii) 2-dose AZI, receive azithromycin twice at a 3-month interval between January and June and placebo twice at a 3-month interval between July and December. AMR, antimicrobial resistance; AZI, azithromycin; LAKANA, Large-scale Assessment of the Key health-promoting Activities of two New mass drug administration regimens with Azithromycin; MDA, mass drug administration

**Fig. 2:**
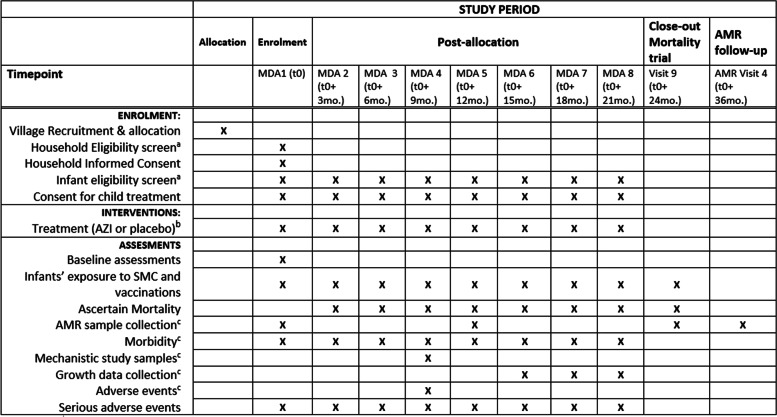
Schedule of enrolment, intervention, and follow-up assessments for the LAKANA trial. ^a^No upper or lower limit in the number of participants per study village, i.e., all households and infants who can be enrolled or treated with study drugs during any MDA visit will be included in the study^. b^Maximum number of treatments given to any single individual 1–11-month-old infant is four. ^c^Data collection on secondary outcomes only in the subset of villages selected for the AMR monitoring. AMR, antimicrobial resistance; AZI, azithromycin; LAKANA, Large-scale Assessment of the Key health-promoting Activities of two New mass drug administration regimens with Azithromycin; MDA, mass drug administration; SMC, seasonal malaria chemoprevention

LAKANA is a cluster-randomized, placebo-controlled, double-blinded, parallel-group, three-arm clinical trial, with an adaptive design comparing the effects on child mortality of azithromycin to placebo administered to 1–11-month-old (age 29–364 days) infants. Participating villages are randomized in a ratio of 3:2:4 to three groups: *placebo* (control); *4-dose AZI*, i.e., azithromycin MDA treatments given every 3 months to 1–11-month-old infants; and *2-dose AZI*, i.e., azithromycin MDA treatments given to 1–11-month-old infants twice at 3-month intervals between January and June, and placebo given twice at 3-month intervals between July and December. The period from January to June corresponds to a season when SMC is not offered by the national malaria control program. The trial design will thus allow testing of the impact of two annual rounds of azithromycin MDA provided quarterly, but only during seasons when SMC is not offered. Villages are recruited for a 24-month follow-up and visited at quarterly intervals, 8 times for study drug administration (MDA visits) and a 9th time for the close-out visit (no MDA, only interviews). In LAKANA, the units of intervention allocation, enrollment to the trial, and treatment with study drugs are different. The unit of intervention allocation (cluster) is the village: in any one village, all infants will receive the same treatment at each MDA visit. The unit of enrollment is a household representative. The unit of treatment with study drugs is an infant: at each MDA visit, each eligible infant in a household will receive study drugs. In consecutive visits, some of the infants will be the same having received study drugs earlier, whereas others will be new—either because they were newly born, they have recently migrated to the area, or they were not available for treatment at the earlier MDA visits. The maximum number of MDA visits in a village over the 24-month follow-up is 8, but the maximum number of treatments (azithromycin or placebo) to any single individual 1–11-month-old infant is 4. Addressing AMR and other study questions requires additional study personnel, equipment, and infrastructure; thus, a subset of villages located closer to the study headquarters and health facilities was selected to form a secondary outcome sample. These villages are to be visited at additional time points, including 1 year after the intervention has stopped. Figure 1 summarizes the mortality trial and AMR study designs. Participant timelines, including the schedule of enrolment, interventions, assessments, and visits, are described in Fig. 2.

### Objectives and hypotheses

#### Primary objective

The primary objective of the LAKANA trial is to determine the impact on mortality of 4 or 2 annual rounds of azithromycin MDA given to 1–11-month-old infants when provided in the context of a national SMC program. Specifically, the aims are to answer the following questions: (i) Does provision of 4 annual rounds of azithromycin MDA to 1–11-month-old infants reduce their mortality in a Mali-like setting? (ii) Does provision of 2 annual rounds of azithromycin MDA to 1–11-month-old infants, when provided quarterly during the non-malaria season, reduce their mortality in a Mali-like setting, in which there is a SMC program? (iii) Does 4 annual rounds of azithromycin MDA to 1–11-month-old infants have a bigger mortality effect than 2 annual rounds of azithromycin MDA? Our hypotheses are (i) 4 annual rounds of azithromycin MDA to 1–11-month-old infants reduce their mortality, (ii) 2 annual rounds of azithromycin MDA to 1–11-month-old infants reduce their mortality, and (iii) 4 annual rounds of azithromycin MDA to 1–11-month-old infants have a higher mortality reduction effect than 2 annual rounds of azithromycin MDA.

As part of the exploratory analysis, we will also assess mortality among children who were 12–59 months old when the latest azithromycin MDA took place in their village of residence. We will also assess effect modification by age at the time of MDA (1–5 months vs 6–11 months) and by sex, anthropometric indicators, seasonality and distribution timing (time since last SMC), cluster level coverage of SMC, cluster level baseline mortality (established at MDA 1), cluster and individual level coverage and the number of administered azithromycin MDA doses, district of residence, distance from the nearest health facility, household asset or income index, and WASH index.

#### Secondary objectives

For AMR monitoring in LAKANA, we aim to address the following study questions: (i) What is the impact of 4 or 2 annual rounds of azithromycin MDA to 1–11-month-old infants on the prevalence of phenotypic azithromycin resistance among *S. pneumoniae* or *E. coli* strains isolated from 4–14-month-old children, who have received 1–4 rounds of azithromycin MDA? (ii) What is the impact of 4 or 2 annual rounds of azithromycin MDA to 1–11-month-old infants on the prevalence of phenotypic azithromycin resistance among *S. pneumoniae* or *E. coli* strains isolated from 49–59-month-old children, who live in the same Malian communities but have not received azithromycin MDA? For the 4–14-month-old children, our hypothesis is that azithromycin does not cause a sustained increase in AMR, i.e., that AMR prevalence will not be higher in the azithromycin clusters than in the control clusters one year after the intervention. For the 49–59-month-old children (who received no MDA), we hypothesize that AMR prevalence will not be higher in the azithromycin clusters than in the control clusters at any point.

As part of an exploratory analysis, we will also compare AMR prevalence in the two azithromycin groups, to investigate if the 4-dose AZI regimen selects for more AMR than the 2-dose AZI regimen. The AMR study procedures are further described in the *AMR* section.

Other secondary objectives of LAKANA include (i) testing a hypothesis that azithromycin MDA to 1–11-month-old infants reduces morbidity for acute infections; (ii) testing a hypothesis that azithromycin MDA to 1–11-month-old infants improves their growth and nutritional status, and (iii) investigating the mechanisms of action of azithromycin, and specifically testing hypotheses that azithromycin MDA eliminates malaria parasitemia and reduces systemic and intestinal inflammation in asymptomatic children.

Finally, in the event of positive clinical results from the LAKANA trial, there is an intention to implement a routine national public health intervention for azithromycin administration in Mali. The feasibility study in LAKANA aims to provide comprehensive policy advice to decision-makers on whether and how this intervention could be implemented (details provided in the “Feasibility” section).

### Study setting and population

LAKANA is being implemented in Mali, a landlocked country located in francophone West Africa. The study headquarters are at the Center for Vaccine Development-Mali (CVD-Mali) in the capital city Bamako. The trial sites consist of rural and peri-urban villages in three regions: Kayes (located in the extreme west, bordering Senegal), Kita (formerly part of Kayes), and Koulikoro (directly east of the Kayes region). Each village in Mali is part of an *aire de santé*, an administrative unit for the purposes of public health management which includes a primary health care facility known as a *Centre de Santé Communautaire* (CSCom); the *aires* are grouped into districts [19]. In villages, community volunteers known as *relais communautaires* serve as a bridge between professional health staff and the villagers [19]. In the defined study area, there are approximately 1.5 million inhabitants, 300,000 of whom are under 5 years old and 60,000 under 1 year old. The three regions are among those with the highest IMR and U5MR in Mali [20]. According to the 2018 demographic and health survey (DHS) report, the IMRs are 69 and 49 deaths per 1000 live births, and U5MRs are 131 and 108 deaths per 1000 live births in Kayes and Koulikoro, respectively [20]. The latest estimates, of 91 deaths per 1000 live births in 2020, place Mali among the 10 countries in the world with the highest mortality rates among children under 5 years of age [1]. Malaria prevalence among 6–59 months is 13% and 22% in Kayes and Koulikoro, respectively [20]. In principle, SMC (with sulfadoxine-pyrimethamine plus amodiaquine) is delivered by the national malaria control program at monthly intervals to children aged 3 to 59 months, up to a maximum of four doses, during the peak malaria transmission season July to October.

### Study development and community engagement

LAKANA was developed in consultation with local, national, and other key stakeholders in child health and survival. Sensitization events with local leaders including regional, district, and village representatives and local healthcare providers are organized by the trial team to discuss the study, seek permissions, and encourage participation in the trial. District and village leaders are consulted for planning the time of randomization events, but they are not otherwise involved in the design of the study. To facilitate activities, the study team is assisted by the community volunteers during study visits to villages. Communities are kept informed about the study progress and meetings are organized as needed, to deal with rumors or any other community anxieties.

### Eligibility criteria

#### Inclusion criteria: cluster

All villages located within Kayes, Kita, or Koulikoro regions, considered non-urban, accessible and safe by the local health authorities and research team, and for which permission from community leadership is granted are eligible for inclusion in the study.

#### Inclusion criteria: household

Households located within a participating village and from which verbal consent (digital signature) from heads of household or an authorized proxy is obtained are eligible for enrolment in the trial.

#### Inclusion criteria: child

All infants residing in a household enrolled in the trial, aged between 29 and 364 days at the time of an MDA, and for whom verbal permission from at least one caregiver is granted, are eligible for treatment with the study drug.

#### Exclusion criteria: child

The exclusion criteria for not receiving the study drug are weight below 3.0 kg (3rd centile for healthy 1-month-old infants in the WHO growth charts) and known allergy to macrolides. At the time of an MDA visit, an infant who is severely ill and is to be referred to a health facility for diagnosis, or an infant who cannot be fed orally, will not receive the study drug.

LAKANA trial participants are advised not to enroll in other clinical trials using antibiotics before the end of the LAKANA follow-up period. However, participation or enrollment in another trial is not an exclusion criterion.

### Interventions

Treatment is provided as a single, oral, directly observed dose of azithromycin (20 mg/kg, weight-based dose) or matching placebo to infants aged 29 to 364 days. The study drugs are packed in non-opaque, plastic bottles. Each bottle contains the same amount of dry powder, either including 1200 mg of azithromycin or respective amount of the base powder. For consumption, a drug bottle is reconstituted with 15 ml of clean commercially bottled water to make 30 ml of study drug suspension. The reconstituted mixture will thus contain either 40 mg/ml azithromycin (active drug) or no azithromycin (placebo). The dose of azithromycin is 0.5 ml/kg child weight, or an equal volume of the respective placebo mixture. At each MDA visit, infants are weighed on an electronic baby hanging scale (ADE Model M111600-01, Hamburg, Germany) as part of the eligibility screening procedure, and the study mobile application automatically calculates the dose in milliliters to be given to the child based on that weight. In practice, infants will receive the following:

- *Control*: placebo mixture every 3 months
- *4-dose AZI*: azithromycin every 3 months
- *2-dose AZI*: azithromycin every 3 months between January and June. Placebo mixture every 3 months between July and December

To ensure adherence, all study drugs are given under direct observation. If an infant has vomited within 15 min of ingesting the study drug, a new similar-size dose is given. All study drugs are donated by Pfizer Inc (New York City, NY, USA) who manufacture, package, and ship them directly to Mali.

### Randomization, allocation concealment, and blinding

The randomization unit is the cluster (village). The randomization is stratified by cluster size, below or above 100 infants per cluster (categorization based on national population estimates). Villages are randomly allocated into the three trial arms at public allocation events where village representatives blindly pull lottery tickets out of a container, each ticket having a two-letter code marked on it.

A total of 18 letters (A, B, C, E, F, G, H, J, K, N, P, R, S, T, U, X, Y, Z) were randomly assigned to represent azithromycin or placebo: 8 letters allocated to azithromycin and 10 letters to placebo. Each village receives two letters: one for visits between January and June and one for visits between July and December. There are 9 possible letter combinations to ensure that treatments are allocated to villages in a ratio of 3:2:4. Of the 9 two-letter combinations: 3 are allocated to control (both letter codes for placebo), 2 for the 4-dose AZI regimen (both letter codes for azithromycin), and 4 for the 2-dose AZI regimen (one letter code for placebo, the other one for azithromycin).

Unmasked personnel (i.e., holders of the trial code) include key members of RTI International; the external partner responsible for data support services at the beginning of the trial, who randomly assigned the treatments to the letters and transmitted the code to Pfizer; key members of Pfizer staff; a statistician external to the trial team; and the Chair of the Data Safety and Monitoring Board (DSMB). Those masked to the study arm allocation are participants, trial investigators including principal investigators (PIs), and site staff including personnel administering treatment and collecting data and samples, and laboratory personnel processing samples for AMR outcomes. Blinding is facilitated by using a matching placebo which is identical to azithromycin in every aspect including appearance, odor, and taste. All study drugs are packaged in identical containers labeled with study-specific labels identical in appearance except for the treatment letter.

The trial code will be opened when all trial data for the 24-month follow-up have been entered into a computer database and data accuracy has been verified. To facilitate an analysis on mortality and other efficacy outcomes while follow-up for AMR is still ongoing, a statistician will temporarily break the code, add group information to a database containing the efficacy outcomes and then recode participant and cluster codes into new identifiers that cannot be linked to the actual identification. With this approach, the statistician can then share a full database with researchers analyzing the efficacy outcomes, while others continuing the data collection of AMR outcomes will remain blinded to the group code. The analysis will first be completed using letter-coded intervention names (A, B, C). Once the PIs have reviewed the results and agreed that there is no need for further data or analysis edits, the code linking treatments to the letter codes will be opened.

### Outcomes and trial assessments

#### Mortality

The primary outcome is the all-cause mortality rate: deaths per 1000 years at risk (PYR) among children who were 1–11-month-old (29–364 days) at MDA. The unit of primary outcome measurement is a 3-month time interval, between successive study visits. The exact dates of the consecutive visits will be used to calculate PYR. Any one child may contribute 1–4 time intervals to the primary outcome analysis.

Mortality is measured via vital status assessment (categorized as alive, died, moved, or unknown) at the quarterly visits. At MDA 1, baseline assessment includes data on births and child deaths in the preceding year to establish U5MR, household residence (GPS coordinates), socio-economic and WASH information, and the demographic data of household members. The infants’ age is calculated based on dates of visit and date of birth obtained primarily from a health card, secondarily from caregivers’ information (if the exact date is known), and tertiarily using a time-bound event calendar. For eligible infants, weight, dose, and the drug code used are recorded along with treatment adherence. Treated infants’ exposure to SMC and Expanded Programme of Immunization (EPI) vaccinations (verified from a health card) are recorded. At subsequent visits (MDA2-8), the vital status of all household’ members is recorded. New members, including newborns, and new households are added. Valid vital status information for infants is obtained from a caregiver who lives in the household. Eligible infants are treated, and treatment information along with SMC and EPI data are recorded. If a child death is reported, the date and cause of death reported by the caregiver are recorded. Cause of death is classified into trauma, acute illness lasting less or more than 2 weeks, or others; no verbal autopsy interview is undertaken. At the close-out visit (visit 9), the vital status of household members and infants’ exposure to SMC and vaccinations will be recorded.

#### AMR

The AMR outcomes include:

- The prevalence of phenotypic and genotypic macrolide resistance among *E. coli* strains isolated from stool samples or *S. pneumoniae* strains isolated from nasopharyngeal swabs among 4–14-month-old children, at 1 year after the MDA intervention has stopped (i.e., 36 months after MDA 1, the baseline)
- The prevalence of phenotypic and genotypic macrolide resistance among *E. coli* strains isolated from stool samples or *S. pneumoniae* strains isolated from nasopharyngeal swabs among 49–59-month-old children, at 24 months after village enrolment to the trial, i.e., after 8 rounds of MDA
- For all isolates of azithromycin-resistant *E. coli* or *S. pneumoniae*, the prevalence of phenotypic and genotypic AMR against antibiotics categorized by the WHO into the “ACCESS” group [21]
- The prevalence of genetic markers of azithromycin and other antibiotic resistance (resistome) of intestinal and nasopharyngeal microbiota among 4–14-month-old children

In a sub-sample of 59 villages (secondary outcome sample) located close to four CSComs near the city of Kita, all 4–14-month-old and 49–59-month-old children are invited to provide biological samples for AMR analysis at baseline (MDA 1) and at 12 months (MDA 5), 24 months (close-out visit), and 36 months after the inclusion of the village into the study. At the time of a visit and before study drug administration (if applicable), one nasal and three rectal swabs from the study infants and children are collected. After collection, the nasopharyngeal swab is placed in STGG media (skimmed milk, tryptone, glucose, and glycerin), one rectal swab is placed in Cary-Blair media, the second rectal swab is placed into DESS media (dimethyl sulfoxide (DMSO)-ethylenediamine tetraacetic acid (EDTA)-saturated salt solution), and the third rectal swab is frozen without preservatives in a cryovial. In villages, the samples are kept in +4°C cooler boxes, then transported within 24–48 h to the main laboratory in Bamako, where a laboratory technician processes and stores them at −80°C. To ensure comparability with national and international AMR statistics, we will primarily study AMR with a traditional phenotypic culture method, isolating individual colonies of selected indicator bacteria (*S. pneumoniae* and *E. coli*) from children and assessing bacterial growth using the disc diffusion method in the presence or absence of azithromycin. We will also employ genetic methods to study microbiota composition and the presence of molecular markers of AMR in host bacteria. As sample collection for the AMR component will take 3 years and molecular methods are rapidly developing, we will store samples for these genetic (and possible other) analyses until the completion of data collection and choose the exact molecular methods only at that time point. Table 1 summarizes the samples taken for AMR analysis.

**Table 1:** Summary of samples taken for antimicrobial resistance analysis

Duration of intervention in community	Child age in months	Biological specimen	Storage media	Estimated number of aliquots	Storage temperature	Number of children
0 months	4–14 and 49–59	Nasopharyngeal swab	STGG media	2	−80°	1350 + 1350
0 months	4–14 and 49–59	Rectal swab	Cary-Blair media	1	−80°	1350 + 1350
0 months	4–14 and 49–59	Rectal swab	DESS media	1	−80°	1350 + 1350
0 months	4–14 and 49–59	Rectal swab	None, stored dry	1	−80°	1350 + 1350
12 months	4–14 and 49–59	Nasopharyngeal swab	STGG media	2	−80°	1350 + 1350
12 months	4–14 and 49–59	Rectal swab	Cary-Blair media	1	−80°	1350 + 1350
12 months	4–14 and 49–59	Rectal swab	DESS media	1	−80°	1350 + 1350
12 months	4–14 and 49–59	Rectal swab	None, stored dry	1	−80°	1350 + 1350
24 months	4–14 and 49–59	Nasopharyngeal swab	STGG media	2	−80°	1350 + 1350
24 months	4–14 and 49–59	Rectal swab	Cary-Blair media	1	−80°	1350 + 1350
24 months	4–14 and 49–59	Rectal swab	DESS Media	1	−80°	1350 + 1350
24 months	4–14 and 49–59	Rectal swab	None, stored dry	1	−80°	1350 + 1350
36 months post-baseline	4–14 and 49–59	Nasopharyngeal swab	STGG media	2	−80°	1350 + 1350
36 months post-baseline	4–14 and 49–59	Rectal swab	Cary-Blair media	1	−80°	1350 + 1350
36 months post-baseline	4–14 and 49–59	Rectal swab	DESS media	1	−80°	1350 + 1350
36 months post-baseline	4–14 and 49–59	Rectal swab	None, stored dry	1	−80°	1350 + 1350

*STGG*, skim milk-tryptone-glucose-glycerin; *DESS*, dimethyl sulfoxide, ethylenediamine tetraacetic acid, saturated salt

In addition to the subset of villages selected in the Kita region, we plan to monitor AMR, and other secondary outcomes, among infants and children living in a subset of villages closer to the capital Bamako, i.e., villages located in Koulikoro or Kati regions (tertiary sample).

#### Mechanisms of azithromycin action, growth, and other health outcomes

Data collection for these analyses will be carried out in the same sub-sample of 59 clusters selected for the AMR monitoring. The outcomes of interest including details on metrics, timeline, and methods of assessment are described in Table 2.

**Table 2:** Summary of secondary outcomes related to mechanisms of azithromycin action, growth, and morbidity

	Biological specimen or measurements	Child age in months	Number of participants	Study visit	Timing of collection versus MDA	Method of assessments
**Mechanism of azithromycin action**						
- Blood malaria parasitemia and hemoglobin concentration - Blood C-reactive protein concentration	Blood	4–11	1000	MDA 4	Paired (before and 14 days after)	From heel prick blood collected, hemoglobin and C-reactive protein concentrations tested with an on-site instrument (QuikRead Go®) Dried blood spots stored for later malaria diagnostics with real-time PCR method
- Fecal neopterin, myeloperoxidase, and alpha-1-antitrypsin concentrations	Stool	4–11	1000	MDA 4	Paired (before and 14 days after)	Fecal neopterin, myeloperoxidase, and alpha-1-antitrypsin concentrations determined with ELISA tests
- Systemic and intestinal inflammation and immune function and development *(future analysis, outcomes to be defined)*	Plasma; peripheral blood mononuclear cells; stool; urine	6–8 and 12–14	1350 + 1350	MDA 6–8 and visit 9	Before	*Storage for future analysis*
**Growth and nutritional status**						
- Mean length-for-age *Z*-score (LAZ) - Mean weight-for-age *Z*-score (WAZ) - Mean weight-for-length *Z*-score (WHZ) - Mid-upper arm circumference *Z*-score (MUAC-Z) - Percentage of moderate or severe stunting (LAZ < −2/LAZ < −3) - Percentage of moderate or severe wasting (WLZ < −2/WLZ < −3)	Length, weight, MUAC	6–8 and 12–14	1800	MDA 6–8	Before	Length assessed using commercial length board (ShorrBoard®, WEIGH AND MEASURE, LLC, Olney, Md, USA) and recorded to the nearest 1 mm. Weight assessed using an electronic infant weighing scale (SECA 354) with reading increments of 10g. MUAC measured with non-stretchable plastic insertion tapes and recorded to the nearest 1 mm. All measurements are done in triplicate
**Morbidity**						
- 14-day period prevalence of fever with respiratory symptoms, fever without respiratory symptoms (proxy for malaria), and diarrhea	Caregiver-reported child morbidity	4–14	*No upper limit*	MDA 1–8 and visit 9	Recall period is 14 days	Caregiver asked if a child has been ill in the preceding 14 days, with fever with respiratory symptoms, fever without respiratory symptoms, diarrhea, or any other symptoms

_*MDA* mass drug administration, *PCR* polymerase chain reaction_

#### Feasibility

The feasibility study framework breaks down into four research components: (i) acceptability, (ii) equity, (iii) health system compatibility, and (iv) economic analysis.

Acceptability is assessed using a mixed methods approach: for collecting qualitative data, we conduct focus-group discussions and semi-structured interviews during the trial implementation with families, and health workers, relais communautaires, village chiefs and elders, and other key community-level stakeholder groups. Additionally, throughout the intervention, we will conduct in-depth interviews with other stakeholders such as health sector decision-makers and politicians. We also collect quantitative data from households through a questionnaire, which covers questions related to both acceptability and the economic analysis, and which is administered in the secondary sample villages.

By collecting various types of background information from the study participants (such as poverty or household location), we will assess the equity of the impact of the intervention. We will also assess the equity of access indirectly, using qualitative interviews among stakeholder groups. The equity data collected will be used when we analyze possible differences in equity outcomes between different implementation strategies we will model.

The health system compatibility study stream will be based on literature and observations on the Malian health system and on interviews with key stakeholders, such as health system researchers, health policy-makers, and health workers at different levels of the system.

The theoretical framework for economic evaluation will be based on standard costing and cost-effectiveness economic evaluation methods. The costing will be conducted from the societal perspective, and it will include economic costs. The effectiveness will be determined by the trial outcome—both the primary mortality outcome and the secondary morbidity and possible AMR effect outcomes. Effectiveness may be expressed in units such as deaths averted or disability-adjusted life years (DALYs).

#### Adverse events

Safety outcomes in the LAKANA trial include:

- Incidence of serious adverse events (SAEs) within 14 days of study drug administration
- Incidence of adverse events (AEs) within 14 days of study drug administration

AEs are defined as any new illness or symptom in the 14 days since MDA, including but not limited to diarrhea, loose stools, vomiting, rash-itching, swelling of the lips, difficulty breathing—wheeze, and crying more than usual. SAEs are defined as any AE experienced by a participant that results in death, is life-threatening, requires inpatient hospitalization or the prolongation of existing hospitalization, or that results in persistent or significant disability or incapacity, or is considered an important medical event by a study physician.

Safety in the LAKANA trial is monitored through passive surveillance at all study sites and active surveillance in the secondary outcome sample. While an MDA is being implemented, the caregivers of all treated infants are instructed to seek medical care and inform the study team or a relais communautaire of any major symptoms experienced within the 14 days following drug administration. CSCom agents are also instructed to report any major events (deaths, hospitalizations) among 1–11-month-old infants recorded within 14 days of an MDA. In addition, 14 days after an MDA in the secondary outcome sample, caregivers of 4–11-month-old infants are interviewed about the child’s experience of AEs. Study coordinators will document and immediately report any suspected SAEs to the study Project Steering Group (PSG), who will inform Pfizer within one business day of the study coordinator’s first awareness of the event. The Malian Institutional Review Board (IRB) will be notified within 72 h of the coordinator becoming aware of the event. Deaths that occur more than 14 days after an MDA or that become known through interviews at subsequent study visits are not considered suspected SAEs. These deaths will be reported as primary outcomes. Participants experiencing an SAE that is likely to be related to the trial intervention will be withdrawn from further study drug provision.

### Sample size

#### Mortality trial

For the primary analyses on mortality, the sample size will be 1150 clusters (villages), or 35,650 infants treated at MDA1, whichever is achieved first. The sample size calculation is based on the following assumptions:

- Mortality among 1–11-month-old children in the control group of 20 deaths per 1000 PYR (20% lower than in the 2018 Mali DHS)
- Mortality in the 2-dose AZI group of 16 deaths per 1000 PYR, i.e., 20% relative risk reduction (RRR). The RRR of 20% is modeled on the MORDOR trial, in which the point estimate for mortality reduction among under-1-year-old infants was 25% [4]. We estimate this reduction to be slightly lower in Mali, because of the SMC that will be offered to 3–11-month-old infants but not offered at the MORDOR sites
- Mortality in the 4-dose AZI of 12 deaths per 1000 PYR, i.e., 40% lower than in the control group and 25% lower than in the 2-dose AZI group. The 40% RRR is also modeled on MORDOR results indicating that practically all effect on mortality was concentrated in the first 3 months after MDA [22].
- 24-month intervention with 8 quarterly rounds of MDA for 50% of clusters, 7 rounds for 30%, and 6 rounds for 20%, due to staggered entry but equal stopping time
- One interim analysis planned, when 60% of the planned 3-month time intervals have been completed
- One-sided 2.5% type 1 error, controlling of multiple group comparisons by the closed testing procedure and multiple looks by the Peto method
- Coefficient of variation (*k*=sd/mean) of 0.1 in mortality among clusters
- Unequal number of infants per cluster: on average 22 infants in a small village and 70 in a big village
- Unequal ratio of clusters. Control vs 4-dose AZI vs 2-dose AZI = 3:2:4

Using the power-by-simulation approach, stratified by the size of the clusters, a sample size of 1150 clusters with on average 31 analyzable infants per cluster per time interval will provide approximately 89% power for testing the hypothesis that biannual azithromycin MDA will reduce mortality, >99% power for testing the hypothesis that quarterly azithromycin MDA will reduce mortality, and 80% power for testing the hypothesis that quarterly azithromycin MDA will reduce mortality more than biannual azithromycin MDA.

#### AMR

The sample size for AMR is 59 clusters or 1350 children per time point. The assumptions used in the sample size calculation are as follows:

- 95% *E. coli* and 65% *S. pneumoniae* recovery rate from the collected samples
- AMR prevalence of 12% in the control group for *E. coli* and 6% for *S. pneumoniae*. These figures come from an analysis of azithromycin resistance in 50 *E. coli* and 50 *S. pneumoniae* samples collected in Mali during the so-called ABCD trial (unpublished observation)
- Non-inferiority margin of 10% points in AMR prevalence
- 80% power, one-sided 2.5% type 1 error rate for each pairwise comparison against a placebo control
- Coefficient of variation of 0.3 in AMR among clusters

### Recruitment and loss-to-follow-up

Villages are planned for recruitment in the trial over a period of 2 years. There is no upper or lower limit to the number of participants per study village, i.e., all households and infants who can be enrolled or treated with study drugs during any MDA visit will be included in the study. At MDA 1, all treated infants will be new to the trial, while on subsequent visits, approximately 25% will be new infants, others having received study drugs earlier. Since each time point will be assessed cross-sectionally, we will not calculate a traditional loss-to-follow-up rate. At each time, we expect to be able to get vital status information from approximately 80% of those receiving study drug MDA at the previous visit. This proportion is based on experience noted in the MORDOR trial, in which there was a 13% loss to follow-up at biannual home visits [4].

### Data collection, management, and monitoring

All data are collected electronically on tablet devices with a custom-designed mobile application (CommCare by Dimagi, Cambridge, MA, USA) and uploaded regularly to a secure, cloud-based server. Real-time monitoring is ensured by error and inconsistency checks at data entry via validation logic rules, mandatory fields, and value range checks. For treatment-related checks, letters assigned to villages after randomization are pre-loaded in the study mobile application. The trial data management team periodically reviews data and resolves queries in consultation with study sites and implements corrections in the database in consultation with the PSG. The full protocol, case report forms (CRFs), and standard operating procedures (SOPs) can be found at the project website at http://lakana.org.

### Statistical methods

#### Statistical analysis

All main analyses will be conducted according to the intention-to-treat principle. The analyses will be conducted blinded as described in the section “Randomization, allocation concealment, and blinding.” The mixed-effects modeling approach will be used to handle the clustered data, with random intercepts representing the clusters. All main analyses will include adjustment for stratification in the randomization scheme as a fixed effect. The analyses will be pre-specified in a Statistical Analysis Plan (SAP), which will be agreed by the trial’s Technical Advisory Group (TAG) prior to conducting the statistical analysis.

The primary outcome analysis will be conducted by a mixed-effect Poisson regression model to estimate the intervention effects on mortality among infants who were 1–11-month-old at the time of receiving the study drug until the next quarterly visit in terms of incidence rate ratio. Duration in days between consecutive quarterly visits will be used as the offset term in the model. As per the close-testing method for controlling multiplicity arising from multiple group comparisons, global null hypothesis of mortality in all three groups being the same will be tested at a 5% significance level. Pairwise null hypothesis can be rejected only if the global null hypothesis has been rejected. Pairwise incidence rate ratio and corresponding 95% confidence intervals will be estimated.

Further details about statistical analysis, including analysis of secondary outcomes, will be available in the separate SAP that will be published later.

#### Interim analysis

**Fig. 3:**
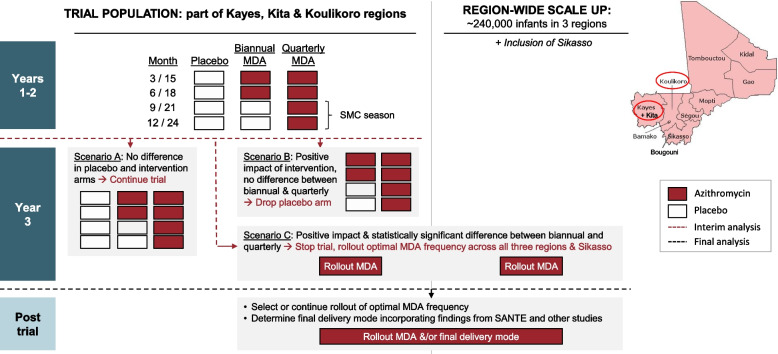
LAKANA trial adaptive design. LAKANA, Large-scale Assessment of the Key health-promoting Activities of two New mass drug administration regimens with Azithromycin; MDA, mass drug administration; SMC, seasonal malaria chemoprevention

A planned interim analysis will be conducted when approximately 60% of the 3-month follow-up intervals have been completed. For this analysis, statistical significance is defined as a 2-sided *p*-value < 0.001 as per the Peto rule [23]. The DSMB statistician will be provided with the material to perform a group-level comparison. Further details about the interim analysis and blind breaking for this analysis will be provided in the SAP. As illustrated in Fig. 3, the conducting of the trial will be altered in the event that the interim analysis gives either of the following results: scenario B—there is statistically significant evidence of a mortality benefit in both azithromycin groups as compared to the placebo group but no statistically significant difference between the two azithromycin groups. In this scenario, the team will drop the placebo arm and re-randomize the previous placebo clusters into either of the two azithromycin groups, at a 1:1 allocation ratio; scenario C—there is statistically significant evidence of a mortality benefit in one or both azithromycin groups and a statistically significant difference between the two azithromycin groups. In this case, the provision of the study MDA will be stopped, and the team will offer to work with the Malian authorities on the implementation of azithromycin MDA in Mali, according to the feasibility study results from the LAKANA trial and from other ongoing projects.

### Study oversight

The PSG is responsible for the overall supervision of the trial. The PSG is composed of the PI and four co-PIs from the participating research institutions: Tampere University in Finland (TAU), CVD-Mali in Mali, the global health consulting company Tro Da Ltd in the UK, and University College London (UCL) in the UK.

Two committees of independent experts, a National Advisory Committee (NAC) consisting of health professionals and the TAG consisting of international experts in the fields of child survival, clinical trials, MDA, and AMR advise on general design issues, implementation strategy, and conduct throughout the life of the trial.

An independent DSMB oversees trial progress and patient safety. The DSMB is composed of members with expertise in pediatrics, infectious disease, epidemiology, and medical statistics. The DSMB meets 1 or 2 times a year. The DSMB convened before the onset of the trial to agree on its terms of reference and on trial stopping rules. The DSMB receives regular summary report of trial progress including documented deviations from the protocol and SAEs. The DSMB will receive the necessary data for the planned interim analysis. The DSMB recommendation to continue or discontinue the trial will not be based on any single issue or value in any of the analyses but on a comprehensive analysis considering multiple issues in and around the trial.

### Ethics and dissemination plan

#### Research ethics approval

The trial is conducted in accordance with the International Conference on Harmonization Good Clinical Practice (ICH-GCP) and adheres to the principles of the Helsinki Declaration and regulatory guidelines in Mali. LAKANA has received approval from the Mali IRB, the Comité d'Éthique de la Faculté de Médecine, de Pharmacie et d’Odonto-Stomatologie (FMPOS), Université des Sciences, des Techniques et des Technologies de Bamako. An ethics committee in Finland has no legal mandate to authorize trials abroad, but the LAKANA trial has received a favorable opinion from the Ethics Committee of the Pirkanmaa Hospital District for Tampere University researchers to participate in the project.

#### Protocol amendments

The PSG makes decisions on amendments to the trial protocol. Any modifications that may have an impact on the conduct of the study are documented in a formal protocol amendment and submitted to the appropriate ethics committees and IRBs for review and approval prior to implementation. The modified protocol is given a new version number and date.

#### Consent

##### Obtaining consent 

The consent process is undertaken by study data collectors*.* All potential participating households in the included villages are given a study information leaflet and shown videos describing the study procedures, in both French and Bambara. The study staff go through the informed consent form (ICF) in detail and answers any questions that arise. The study staff then as ask for consent from the head of household or his/her representative to enroll the household in the trial, including the eight MDA treatments and the 24-month follow-up. The response is given verbally and documented electronically in the study mobile application. If the person giving consent is illiterate, an impartial witness of the person’s choice is invited to witness the procedure. Verbal consent from a caregiver is required, at each MDA, prior to administering the study drug to an eligible infant. In the subset of villages selected for the AMR study, information about participation in the AMR and other sub-studies is given to household representatives. Written informed consent from these representatives is obtained for children who are to be invited for further data and biological sample collection. This consent is documented electronically on the study mobile application and the signed paper copy of the ICF is given to the household representative for their records (see Additional file 1 for the informed consent materials).

##### Ancillary studies

Information and ICF relating to participation in the AMR and other sub-studies include consent for sample collection, storage, and analysis at CVD-Mali laboratory in Bamako, as well as for shipping to collaborating centers in the UK, Finland, and possibly elsewhere for later laboratory analysis relating to other hypotheses, to be determined once the mortality effect is known and ongoing mechanistic studies have been completed. Any future research will undergo ethical review.

#### Compensation

For most LAKANA participants, the nine scheduled home visits will take approximately 20–30 min, and households will not be compensated. Children in the secondary outcome sample and from whom blood is drawn will be offered something to eat and drink.

#### Confidentiality

Access to the tablet computers and the cloud-based data is username and password-protected. During transmission and in the cloud, data are encrypted to ensure data privacy. The tablet computers are stored and charged overnight at locked study offices. Once enrolled, participants are assigned a quick response (QR) code that contains a unique identifier. All documentation regarding participants, including laboratory samples, source data, and CRFs, are identified in the database with these identifiers. Names appear on ICFs and a separate coding list. A minimal set of identifying information, necessary for the implementation of the trial, is stored in an encrypted form on the data collection devices and in a separate encrypted database. Access to the identifying information is limited to authorized data collectors with a secure username and password. Clinical information will not be released without the written permission of the subject, except as required for monitoring purposes.

#### Access to data

De-identified data collected, as well as respective metadata, are prospectively uploaded to a suitable non-profit website, https://clinepidb.org, and will be made publicly available at the end of the trial.

#### Ancillary and post-trial care

All medical costs for study participants, incurred as a result of their participation in the LAKANA trial, will be covered by clinical trial insurance purchased by the trial team. Medical costs due to adverse reactions arising during the study will be covered by the study budget. At the end of the trial, if the analysis suggests a reduction in mortality attributed to azithromycin, all 1–11-month-old infants in participating households will be offered a 20-mg/kg dose of azithromycin free of cost. If deemed appropriate thereafter, the study team will work with the Malian authorities to roll out a longer-term azithromycin MDA program at the trial site and elsewhere in Mali.

#### Dissemination policy

##### Plans

The results of this study will be presented at local, national, and international meetings. The results will be distributed and discussed with key stakeholders, including the Malian Ministry of Health (MOH), the WHO, and relevant organizations that implement child health policies. The results will be published in peer-reviewed journals in open-access format per funder guidelines.

##### Authorship

Authorship will be based on the International Committee of Medical Journal Editors (ICMJE) guidelines. The PSG considers all proposed publications and will have the final decision on content and authorship. Group authorship may be attributed where appropriate.

#### Monitoring and auditing

External monitoring is provided by an independent clinical research organization (CRO), Likak Research, which oversees the progress of the trial ensuring that it is conducted, recorded, and reported in accordance with the approved protocol, SOPs, GCPs, and applicable regulatory requirements. Access to the Trial Regulatory File, ICFs, laboratory records, periodic data descriptives, and queries is granted to the CRO for monitoring purposes. The CRO completed a pre-site trial assessment, conducts quarterly monitoring visits during the trial implementation, and will complete a data collection closure visit.

## Discussion

Encouraging findings from trachoma control interventions delivering azithromycin MDA, supported by results from large trials that followed, notably MORDOR [4], led to mass administration of azithromycin being considered a promising strategy for promoting child survival in high-mortality settings. However, various questions persist, and further evidence of efficacy needs to be demonstrated to inform policy on azithromycin MDA to reduce child mortality. Along with other ongoing trials, such as the Community Health Azithromycin Trial (CHAT) [24], the Infant Mortality Reduction by the Mass Administration of Azithromycin (MIRAMA) (ClinicalTrials.gov NCT04716712) in Burkina Faso, and Azithromycin for Child Survival in Niger: Mortality and Resistance (AVENIR) [25], LAKANA will contribute to pending discussions about efficacy, optimal frequency of MDA and timing around SMC, AMR, and azithromycin’s mechanisms of action. In Mali, the results of our study will complement those of other trials testing alternative methods of azithromycin delivery such as Oral Azithromycin to Prevent Stillbirths and Infant Mortality (SANTE) (ClinicalTrials.gov NCT03909737), which is evaluating the benefits of azithromycin provided to pregnant women and their infants at the 6-week EPI visit.

Treatment of latent or subclinical infections is often hypothesized as the pathway leading to a mortality benefit among communities exposed to azithromycin MDA. In the Niger site of the MORDOR trial, azithromycin MDA was associated with reduced prevalence of malaria parasitemia, and verbal autopsies revealed fewer deaths from malaria, dysentery, meningitis, and pneumonia in communities treated with azithromycin [26]. We will build on this work and investigate in a subset of villages, the effect of azithromycin on malaria parasitemia, inflammation, and other markers of infection, immunity, and vaccine response. Besides the infection reduction pathway, growth promotion is considered another plausible mechanism that could explain some of the mortality benefit [27]. Undernutrition is an underlying factor contributing in almost half of all child deaths [1], and in low- and middle-income settings, antibiotic treatment of children might improve their linear growth [28]. In LAKANA, we will investigate the impact of azithromycin MDA on infants’ growth and nutritional status.

Monitoring and documenting potential harms in the context of a mass treatment use of antibiotics is critical. Azithromycin has an excellent safety profile, with mostly mild adverse effects related to gastrointestinal symptoms [29]. A rare yet possible SAE is the increased risk of infants aged less than 28 days developing pyloric stenosis [30, 31]. The use and safety of azithromycin in neonates is under investigation in the Neonates and Azithromycin, an Innovation in the Treatment of Children in Burkina Faso (NAITRE) trial (ClinicalTrials.gov NCT03682653). Based on current evidence, infants under 29 days of age are excluded from treatment provision in LAKANA, and both a passive and active surveillance system will allow us to closely monitor AEs in 1–11-month-old infants treated. In addition, monitoring AMR is a crucial component of LAKANA. We will assess the prevalence of resistance to macrolides and other antibiotic classes, before, during, and after the intervention both among treated infants and their communities and examine the stability of any increase post-study cessation. In MORDOR, there was an increase in the prevalence of macrolide resistance in pneumococcal isolates [17]. In communities at the Malawi site, significant changes in the AMR profile and gut microbiome were reported after four biannual rounds of azithromycin [32]. Other studies have also reported macrolide resistance after regular azithromycin MDA [33–35]. However, these results are challenged by existing findings showing no evidence of a rise in AMR after one or multiple MDA rounds [36], or evidence of a quick decline after discontinuation of treatment [37]. The WHO recommends monitoring the rate of AMR occurrence in both nasopharyngeal and gut flora at the community level for all antibiotics in their Essential Medicine List. With the development of long-read sequencing technologies, such as nanopore sequencing, the ability to track AMR genes within bacterial populations is accessible. We can now identify the specific genes conferring resistance as well as whole genome sequencing for the detection of all AMR genes present. Metagenomic sequencing can enable a universal picture of the “resistome” and can identify how mass drug administration affects the microbiota. As there are many studies trialing mass drug administration and monitoring AMR, it is important that AMR screening methods are (i) feasible and optimized for use in a resource-limited setting, (ii) validated against standard phenotypic techniques, and (iii) standardized across clinical trials in order to pool results and compare findings. These requirements are currently being addressed as part of this study.

If proven to be effective, the acceptability and feasibility aspects of providing mass treatment azithromycin to infants with no clinical indication will be critical in determining whether to adopt and scale up such strategies for routine use. Understanding the perspectives and views of various stakeholders on MDA intervention and its implementation will thus be vitally important. The feasibility study in the LAKANA trial will inform on ways in which stakeholders conceptualize, perceive, and experience the intervention, and on possible implementation strategy given the context of Mali.

## Conclusion

The LAKANA trial may offer a partial solution to successfully reduce the high child mortality in Mali and elsewhere in sub-Saharan Africa. The combined results of our study and of the other ongoing trials may be decisive in enabling evidence-based public health recommendations to be made about the optimal use of azithromycin for child survival. In Mali, if LAKANA provides evidence in support of a positive mortality benefit resulting from azithromycin MDA, the study will form the first phase of a national public health program.

## Trial status

This manuscript refers to protocol version 4.0—27 June 2022. Enrolment commenced on October 15, 2020, and is planned to be completed by the end of 2022. The expected final data collection date for the primary outcome measurement is June 2024, while the study is expected to be completed in December 2024. The trial was registered on 11 June 2020 at ClinicalTrials.gov under ID: NCT04424511 (see Additional file 2 for the World Health Organization Trial Registration Data Set). We used the Standard Protocol Items: Recommendations for Interventional Trials (SPIRIT) checklist when writing the current report [38] (Additional file 3).

## Supplementary Information

**Additional file 1.** Informed consent materials.

**Additional file 2.** World Health Organization Trial Registration Data Set.

**Additional file 3.** Reporting checklist for protocol of a clinical trial.

## Data Availability

Not applicable. Upon completion of the trial, de-identified data will be made publicly available per the funder, the Bill and Melinda Gates Foundation, policy.

## Abbreviations

AE: Adverse event
AMR: Antimicrobial resistance
ARI: Acute respiratory infections
AZI: Azithromycin
CRF: Case report form
CRO: Clinical research organization
CRP: C-reactive protein
CSCom: Centre de Santé Communautaire
CVD-Mali: Center for Vaccine development-Mali
DALYs: Disability-adjusted life years
DHS: Demographic and health survey
DSMB: Data Safety and Monitoring Board
EPI: Expanded Programme of Immunization
GCP: Good Clinical Practice
ICF: Informed consent form
ICMJE: International Committee of Medical Journal Editors
IRB: Institutional Review Board
LAKANA: Large-scale Assessment of the Key health-promoting Activities of two New mass drug administration regimens with Azithromycin
LAZ: Length-for-age *Z*-score
MDA: Mass drug administration
MOH: Ministry of Health
MORDOR: Macrolides Oraux pour Réduire les Décès avec un Oeil sur la Résistance
MUAC: Mid-upper arm circumference
NAC: National Advisory Committee
PIs: Principal investigators
PYR: Person-years at risk
QR: Quick response
RR: Relative risks
SAE: Serious adverse event
SAP: Statistical Analysis Plan
SDG: Sustainable development goal
SMC: Seasonal malaria chemoprevention
SOP: Standard operating procedure
SPIRIT: Standard Protocol Items Recommendations for Interventional Trials
SSA: Sub-Saharan Africa
TAG: Technical Advisory Group
U5MR: Under-5 mortality rate
WASH: Water, sanitation, and hygiene
WAZ: Weight-for-age *Z*-score
WLZ: Weight-for-length *Z*-score
