# Supplementary material for: Testing the effects of mass drug administration of azithromycin on mortality and other outcomes among 1–11-month-old infants in Mali (LAKANA): study protocol for a cluster-randomized, placebo-controlled, double-blinded, parallel-group, three-arm clinical trial
Source: Trials. 2023 Jan 3;24:5. doi: 10.1186/s13063-022-06966-7 (PMC9809521; doi:10.1186/s13063-022-06966-7)
Supplement: Supplementary file 1 — Additional file 1. Informed consent materials. [file 13063_2022_6966_MOESM1_ESM.pdf]

12. Appendices

Appendix 1a: Information about the trial to village leadership

(English version, to be translated into French and Bambara and read aloud to village leaders

CENTRE POUR LE DEVELOPPEMENT DES VACCINS, MALI

Director General: Samba Ousmane SOW, M.D., M.Sc., FASTMH

Professor of Medicine

Infectious Diseases Project: Measles Vaccine Initiative ♦ Enteric Disease Project ♦ Pneumococcus, Meningococcus, & *Haemophilus influenzae* Prevention Program
Malaria Project: Bandiagara Malaria Project ♦ Malaria Training Program

CVD-MALI

Large-scale Assessment of the Key health-promoting Activities of New mass drug administration regimens with Azithromycin (LAKANA)

Information for Participants and Informed Consent Forms for village leaders

This document contains information for villages participating in a cluster-randomised, double-blinded, parallel group, controlled trial, to test the effects of mass drug administration of azithromycin on mortality and other outcomes among 1-11-month-old infants in rural Mali. It also contains the necessary consent forms for participants to enrol in the trial.

Key Investigators

Centre pour le Développement des Vaccins–Bamako, Mali

Per Ashorn, MD, PhD [1], Ulla Ashorn, PhD [1], Yin Bun Cheung, PhD [1,2], Camilla Ducker, MSc., MBBS [3], Nigel Klein, MBBS, PhD [4], Samba O. Sow MD., MSc., FASTMH [5].

Participating Academic Institutions

[1] Tampere University, Finland; [2] Duke-NUS Medical School, Singapore; [3] Tro Da Ltd, UK; [4] University College London, UK; [5] CVD-Mali, Bamako, Mali.

Protocol version 4.0: 27. June 2022

Study Overview

Your village is invited to participate in the LAKANA trial, in which we will try to determine if a drug called azithromycin can help Malian infants to enjoy better health and better rates of survival. In the study, some infants will receive azithromycin and others will receive something called a placebo, which looks and tastes like azithromycin but does not contain any medicine. The study will compare these different groups of children.

Azithromycin is an effective and safe medicine that is commonly used to treat illnesses – it has been used for many years to successfully and safely treat infections, including millions of cases of an eye infection called trachoma. There are studies that show that periodically using this medicine to treat healthy children in West Africa might reduce mortality. The LAKANA trial team is investigating if such treatment would also be beneficial to Malian infants.

In total, we plan to include 50–60 000 children in the study, from approximately 1150 villages or urban areas in the Kayes, Kita and Koulikoro regions. If you give consent for your village to participate, the LAKANA team will visit the village every three months over the next two years, to ask you some questions, and give study medicine to all 1-11 month old infants living in the village.

Occasionally, children who take azithromycin may suffer stomach upsets for a short period, but otherwise we don't expect the treatment to cause any harmful side effects. Reactions, although rare, are always possible when medicines are given to children. So we will monitor the health of all the children who receive the medicine. In the unlikely case that a child in your village experiences a negative reaction, CVD-Mali will help that child to receive medical care and cover any costs related to that care.

This study could lead to major improvements in the health of infants in Mali. By participating in this trial, your village may be contributing to the development of better health programmes for the whole country. If this trial is successful, it is possible that azithromycin may be given to infants across Mali and West Africa.

You do not have to give permission for your village to take part in this study – you are free to decide if the village should participate or not.

What is the study about?

Azithromycin is a type of medicine, an antibiotic, that is commonly used to treat infections, including eye infections that cause blindness. Recent research studies have shown that infants in certain African countries had better survival rates when they took azithromycin every six months, when compared to those who did not take the medicine. In the LAKANA trial, researchers from the Centre pour le Développement des Vaccins-Mali (CVD-Mali) and their partners in Finland and the United Kingdom are studying how well azithromycin works in Mali. The trial is being funded by an American organization called the Bill & Melinda Gates Foundation.

The LAKANA trial will be carried out in 1150 villages and urban areas in the Kayes, Kita,

and Koulikoro regions, if local representatives have given permission for study activities to take place there. After receiving permission, in each village or town area, the study team will visit all households every three months for a period of two years. The team members will ask for permission to record and store a list of household members. If there are any 1-11 month old infants in the household, the household will be offered the chance to participate in the study and the infants living there will be offered the study medicine. At subsequent visits, team members will monitor the effects of the study medicine by asking questions about the infants' health.

Two types of study mixture will be given to infants in the LAKANA trial: azithromycin and placebo. Placebo is something that looks and tastes like azithromycin but does not contain any active ingredient that can treat infection. In one third of the study villages, all infants will receive azithromycin during all the study visits, every three-months. In another third of the study villages, infants will receive azithromycin at half of the visits and placebo at the other half. In the last third of the study villages, infants will always receive placebo.

Deciding which villages would be in which group was done by chance, like taking one single grain from a bag full of rice. During the study nobody will know whether the infants in your village are receiving placebo or active azithromycin at any of the home visits – this information will only be available later on to the study team. This way of carrying out the trial will help the study team to make correct conclusions about the health effects of azithromycin.

Study outcomes

The study will test to see if azithromycin has a positive impact on infant health and if it reduces infant mortality. The results of the study will be used to influence public health policy in Mali and in the wider sub-Saharan African region.

At approximately midway during the trial, researchers will analyze the obtained results. If there are clear indications that azithromycin is having a positive effect on mortality and the general health of infants, the trial will be modified, and azithromycin will then be given to all infants in the selected areas – and in other regions of Mali.

Who can take part in this study?

All households in the villages selected for the study will be offered the chance to join the study, for the purposes of collecting data. At each of the quarterly visits to households taking part in the study, all 1-11 month-old (age 29-364 days) infants will be offered the study medicine, as long as the infant has no known intolerance to azithromycin.

What will happen in the study?

If your village takes part in this study, the study team will visit the village four times a year for two years (eight times in total, after an initial visit to collect information). At each visit, we will ask participants questions about their households and their infants' health and illnesses, we will measure the weight of 1-11 month old infants, and will give them an appropriate dose (one teaspoonful or less) of study medicine. The medicine will

come in the form of a mixture and will be placed in the infant's mouth using a disposable syringe.

Since the study visits will take place at three-month intervals and only 1-11 month old (age 29-364 days) infants will be treated, the maximum number of treatments given to any individual infant will be four.

The first visit will take approximately 45 minutes, the subsequent visits will likely take approximately 15 minutes each.

If any household or child enrolled in the study is not at home when the study team comes to visit, they will try to visit again later or they may call to agree on a time when the missing people will be at home. If for some reason the visit cannot be completed, infants can still continue to take part in the study and will be seen during subsequent home visits.

How do I give consent for trial participation?

As representatives of your village, you will be asked for permission to carry out trial-related activities in the village. If we receive your permission, we will start the study activities. First of all, at each household, we will ask for permission to visit the household and ask some questions every three months for the next two years. Additionally, on each visit, we will ask for parents' or guardians' permission to weigh 1-11 month old infants and administer study medicine to them.

You don't need to sign any documents to give consent for the trial to take place in your village but we will record your consent on our computer. We will also ask an independent person to witness your consent if you are not literate. His or her name will also be recorded in the computer system.

Is participation voluntary?

Your village's participation in this study is entirely voluntary – for the village as a whole and for each individual household/child within the village. Your village does not have to take part in this study. Once enrolled, individual participants in the study will be free to leave the study at any time without giving a reason. Infants' participation will not change anything about the health care they receive at any health facilities. If, for any reason, infants are no longer available for follow-up visits, we will ask to contact them again to check on their health. Participants may refuse this as well. If a participant withdraws from this study, we will keep the information that we have already collected from infants in your village.

We will tell you about any significant new findings that are available during the study that may affect your willingness to involve infants in your village in the study.

Can villages / infants be removed from this study?

The study doctors and Malian, Finnish, or UK-based research committees overseeing the safety and rights of study participants, can remove your village and/or any infants in the village from the study or even stop the entire study without your approval if serious

health problems are noticed. If a parent or guardian decides to stop an infant's participation in the study, or if an infant's participation is ended, the study doctor or study staff may still ask the parents/guardian about that infant's participation in the study. Answering any such questions is, however, voluntary.

What are my village's responsibilities if we take part in this study?

If you agree to your village's participation in this research, we will visit households every three months and ask villagers to inform us if they move or expect to be unavailable. If villages and individuals agree to take part Taking part in this study does not change responsibilities with regard to other non-study related health care for infants in the village.

What risks are involved in azithromycin use and trial participation?

Azithromycin is commonly used and is known to be safe for infants. Even so, a few people may react to azithromycin and experience brief episodes of diarrhoea, nausea, abdominal pain and vomiting. A very small number of children may also experience liver problems, rashes, itching, or swelling of the lips or throat when they take azithromycin.

In extremely rare cases, some young infants may experience a problem where they have trouble passing food from the stomach to the intestines, which can cause vomiting and may require surgery. This can happen to any infant, whether they take azithromycin or not. If an infant experiences a reaction like this or has developed other symptoms, care should be sought urgently at the closest health facility and the study doctor should also be contacted.

If an infant in your village falls ill because of the study treatment, CVD-Mali will pay for the costs of medical treatment according to Malian standards of care.

What are the possible benefits of being in this study?

There may be no direct benefits to the people in your village as a result of participating in this study. However, the information obtained in this study will be important in deciding if azithromycin could improve the health of infants in Mali and other similar countries.

What kind of feedback can we expect from research results?

The results of the study will be known after it is finished. The study team will hold meetings to inform the community about the results.

What are the costs of taking part in this study?

All research activities will be performed free of charge.

What are the payments for being in this study?

There are no payments for taking part in the study. But infants in your village will be visited every three months while the study is active, and participants may signal other issues related to infants' health to the *relais* and data collector who will visit you.

What are the alternatives to participating in this study?

Giving azithromycin to infants in Mali is not currently part of routine care. The alternative to participating in the study is choosing not to take part.

How will information be kept private?

To protect the privacy of the parents/guardians and children living in your village, we will keep the information collected by the study secure. We will only allow authorized people to see it, including the study team from Mali, UK, and Finland, their representatives and the Ethics Committee in Mali, who act to protect people who take part in research. If study results are released to the public, the identities of participants will not be shared.

Who can answer questions about this study?

If you have any questions regarding your infant's participation or if an infant has a problem as a result of participating in this study, you may contact at any time Dr Fatoumata Diallo on (00223) 74 60 18 19, Dr Adama Coulibaly on (00223) 66 05 04 16, Dr Fadima Cheick Haidara on (0023) 66 73 34 91, Prof Samba Sow on (00223) 76348947, or CVD-Mali, CNAM, Ex-Institut Marchoux on (00223) 20 23 60 31.

To learn more about the ethical approval of this study or about your rights as a research subject, you can contact the Ethics Committee of FMPOS at (00223) 2022 5277, Prof. Mamadou Marouf Keita on (00223) 66722022 or Prof. Mahamadou Diakité (Permanent Secretary) at (00223) 66231191

Appendix 1b: Verbal Consent form for Village Participation

To be recorded electronically by a member of the LAKANA study team, on behalf of the head of the village chiefs, representatives or other persons authorised to speak on behalf of a village/grouping of hamlets

The giving and recording of consent should be confirmed by an independent witness

The persons named below affirm that they have given verbal consent and received information about the proposed trial or had this information explained to them, and that they consent to all eligible infants living in their village being enrolled in the trial. Witnesses to the consent process affirm that consent was given and information about the trial received according to the conditions laid out in the information about the trial.

Name of village

____

Name of Person authorised to give consent on behalf of village

____

Position / Role

Did the above-named person give consent? Yes/No Date

Name of Witness to Consent Procedures: ______________________________

(*If subject is illiterate, or otherwise unable to give consent)*

____

Witness's Confirmation Yes/No Date

Name of Investigator: ______________________________

or Authorized Representative obtaining informed consent

____

Investigator's Confirmation (initials or study code) Yes/No Date

Appendix 2a: Information about the trial to a potential participant

(English version, to be translated into French and Bambara and read aloud to village leaders

CENTRE POUR LE DEVELOPPEMENT DES VACCINS, MALI

Director General: Samba Ousmane SOW, M.D., M.Sc., FASTMH

Professor of Medicine

Infectious Diseases Project: Measles Vaccine Initiative ♦ Enteric Disease Project ♦ Pneumococcus, Meningococcus, & *Haemophilus influenzae* Prevention Program
Malaria Project: Bandiagara Malaria Project ♦ Malaria Training Program

CVD-MALI

Large-scale Assessment of the Key health-promoting Activities of New mass drug administration regimens with Azithromycin (LAKANA)

Information for Participants and Informed Consent Forms for households

This document contains information for participants in a cluster-randomised, double-blinded, parallel group, controlled trial, to test the effects of mass drug administration of azithromycin on mortality and other outcomes among 1-11-month-old infants in rural Mali. It also contains the necessary consent forms for participants to enrol in the trial.

Key Investigators

Centre pour le Développement des Vaccins–Bamako, Mali

Per Ashorn, MD, PhD [1], Ulla Ashorn, PhD [1], Yin Bun Cheung, PhD [1,2], Camilla Ducker, MSc., MBBS [3], Nigel Klein, MBBS, PhD [4], Samba O. Sow MD., MSc., FASTMH [5].

Participating Academic Institutions

[1] Tampere University, Finland; [2] Duke-NUS Medical School, Singapore; [3] Tro Da Ltd, UK; [4] University College London, UK; [5] CVD-Mali, Bamako, Mali.

Study Overview

You are invited to participate in the LAKANA trial, in which we will try to determine if a drug called azithromycin can help Malian infants to enjoy better health and better rates of survival. In the study, some infants will receive azithromycin and others will receive something called a placebo, which looks and tastes like azithromycin but does not contain any medicine. The study will compare these different groups of children.

Azithromycin is an effective and safe medicine that is commonly used to treat illnesses – it has been used for many years to successfully and safely treat infections, including millions of cases of an eye infection called trachoma. There are studies that show that periodically using this medicine to treat healthy children in West Africa might reduce mortality. The LAKANA trial team is investigating if such treatment would also be beneficial to Malian infants.

In total, we plan to include 50–60 000 children in the study, from approximately 1150 villages or urban areas in the Kayes, Kita, and Koulikoro regions. If you agree to participate, the LAKANA team will visit you every three months over the next two years, to ask you some questions, and give study medicine to 1-11 month old infants living in your household.

Occasionally, children who take azithromycin may suffer stomach upsets for a short period, but otherwise we don’t expect the treatment to cause any harmful side effects. Reactions, although rare, are always possible when medicines are given to children. So we will monitor the health of your child(ren) after they receive the medicine. In the unlikely case that your child experiences a negative reaction, CVD-Mali will help the child to receive medical care and cover any costs related to that care.

This study could lead to major improvements in the health of infants in Mali. By participating in this trial, you may be contributing to the development of better health programmes for the whole country. If this trial is successful, it is possible that azithromycin may be given to infants across Mali and West Africa.

You do not have to take part in this study – you are free to decide if you want to participate or not.

What is the study about?

Azithromycin is a type of medicine, an antibiotic, that is commonly used to treat infections, including eye infections that cause blindness. Recent research studies have shown that infants in certain African countries had better survival rates when they took azithromycin every six months, when compared to those who did not take the medicine. In the LAKANA trial, researchers from Centre pour le Développement des Vaccins-Mali (CVD-Mali) and their partners in Finland and the United Kingdom are studying how well azithromycin works in Mali. The trial is being funded by an American organization called the Bill & Melinda Gates Foundation.

The LAKANA trial will be carried out in 1150 villages or urban areas in the Kayes, Kita,

and Koulikoro regions, after local representatives have given permission for study activities to take place there. In each village or town area, the study team will visit all households every three months for a period of two years. The team members will ask for permission to record and store a list of household members. If there are any 1-11 month old infants in the household, the household will be offered the chance to participate in the study and the infants living there will be offered the study medicine. At subsequent visits, team members will monitor the effects of the study medicine by asking questions about the infants' health.

Two types of study mixture will be given to infants in the LAKANA trial: azithromycin and placebo. Placebo is something that looks and tastes like azithromycin but does not contain any active ingredient that can treat infection. In approximately one third of the study villages, all infants will receive azithromycin during all the study visits, every three-months. In another third of the study villages, infants will receive azithromycin at half of the visits and placebo at the other half. In the last third of the study villages, infants will always receive placebo.

Deciding which villages would be in which group was done by chance, like taking one single grain from a bag full of rice. During the study neither you nor anyone in the study team will know whether your infant receives placebo or active azithromycin at any of the home visits – this information will only be available later on to the study team. This way of carrying out the trial will help the study team to make correct conclusions about the health effects of azithromycin.

Study outcomes

The study will test to see if azithromycin has a positive impact on infant health and if it reduces infant mortality. The results of the study will be used to influence public health policy in Mali and in the wider sub-Saharan African region.

At approximately midway during the trial, researchers will analyze the obtained results. If there are clear indications that azithromycin is having a positive effect on mortality and the general health of infants, the trial will be modified, and azithromycin will then be given to all infants in the selected areas – and in other regions of Mali.

Who can take part in this study?

All households in the study area will be offered the chance to join the study for the purposes of collecting data. At each of the quarterly visits to households taking part in the study, all 1-11 month old infants will be offered the study medicine, as long as the infant has no known intolerance to azithromycin.

What will happen in the study?

If you take part in this study, the study team will visit you four times a year for two years (eight times in total, after an initial visit to collect information). At each visit, we will ask you some questions about your household and your infants' health and illnesses, we will measure the weight of your 1-11 month old infants, and will give them an appropriate dose (one teaspoonful or less) of study medicine. The medicine will come in the form of a mixture and will be placed in the infant's mouth using a disposable syringe.

Since the study visits will take place at three-month intervals and only 1-11 month old infants will be treated, the maximum number of treatments given to any individual infant will be four.

The first visit will take approximately 45 minutes of your time, the subsequent visits will likely take approximately 15 minutes each.

If you are not at home when the study team comes to see you, they will try to visit again later or they may call you to agree on a time when you will be at home. If for some reason the visit cannot be completed, you and your infant can still continue to take part in the study and be seen during subsequent home visits.

How do I give my consent for trial participation?

Before starting any trial-related activities in your home village, we will have received permission to conduct trial activities there from your village representatives. At your household, first of all, we will ask for permission to visit you and ask some questions every three months for the next two years. Additionally, on each visit, we will ask your permission to weigh your 1-11 month old infants and administer study medicine to them.

You don’t need to sign any documents for your participation, but we will record your consent on our computer. We will also ask an independent person to witness your consent if you are not literate. His or her name will also be recorded in the computer system.

Is participation voluntary?

Your infant’s participation in this study is entirely voluntary. You do not have to take part in this study, and you are free to leave the study at any time without giving a reason. Your infant’s participation will not change anything about the health care your infant receives at any health facilities. If, for any reason, you are no longer available for follow-up visits, we will ask to contact you again to check on the health of your infant. You may refuse this as well. If you withdraw from this study, we will keep the information that we have already collected from your infant.

We will tell you about any significant new findings that are available during the study that may affect your willingness to involve your infant in the study.

Can my infant be removed from this study?

The study doctors and Malian, Finnish, or UK-based research committees overseeing the safety and rights of study participants, can remove you or your infant from the study or even stop the entire study without your approval if serious health problems are noticed. If you decide to stop your infant’s participation in the study, or if your infant’s participation is ended, the study doctor or study staff may ask you some questions about your infant’s participation in the study. Answering any such questions is, however, voluntary.

What are my responsibilities if I take part in this study?

If you participate in this research, we will visit your household every three months and

ask you to inform us if you move or expect to be unavailable. If you give consent for your infant to participate in this study, you will still be responsible for all other non-study related health care for your infant.

What risks are involved in azithromycin use and trial participation?

Azithromycin is commonly used and is known to be safe for infants. Even so, a few people may react to azithromycin and experience brief episodes of diarrhoea, nausea, abdominal pain and vomiting. A very small number of children may also experience liver problems, rashes, itching, or swelling of the lips or throat when they take azithromycin.

In extremely rare cases, some young infants may experience a problem where they have trouble passing food from the stomach to the intestines, which can cause vomiting and may require surgery. This can happen to any infant, whether they take azithromycin or not. If you think your infant is experiencing a reaction like this or has developed other symptoms, please seek care urgently at the closest health facility and contact the study doctor.

If your infant falls ill because of the study treatment, CVD-Mali will pay for the costs of medical treatment according to Malian standards of care.

What are the possible benefits of being in this study?

There may be no direct benefits to you or your infant as a result of participating in this study. However, the information obtained in this study will be important in deciding if azithromycin could improve the health of infants in Mali and other similar countries.

What kind of feedback can I expect from research results?

We will know the results of the study after it is finished. The study team will hold meetings to inform the community about the results.

What are the costs of taking part in this study?

All research activities will be performed free of charge.

What are the payments for being in this study?

There are no payments for taking part in the study. But you and your infant will be visited every three months while the study is active, and you may signal other issues related to your infant's health to the *relais* and data collector who will visit you.

What are the alternatives to participating in this study?

Giving azithromycin to infants in Mali is not currently part of routine care. The alternative to participating in the study is choosing not to take part.

How will information be kept private?

To protect your privacy, we will keep the information collected by the study about your infant secure. We will only allow authorized people to see it, including the study team

from Mali, UK, and Finland, their representatives and the Ethics Committee in Mali, who act to protect people who take part in research. If study results are released to the public, the identities of participants will not be shared.

Who can answer questions about this study?

If you have any questions regarding your infant's participation or if your infant has a problem as a result of participating in this study, you may contact at any time Dr Fatoumata Diallo on (00223) 74 60 18 19, Dr Adama Coulibaly on (00223) 66 05 04 16, Dr Fadima Cheick Haidara on (0023) 66 73 34 91, Prof Samba Sow on (00223) 76348947, or CVD-Mali, CNAM, Ex-Institut Marchoux on (00223) 20 23 60 31.

To learn more about the ethical approval of this study or your rights as a research subject, you can contact the Ethics Committee of FMPOS at (00223) 2022 5277, Prof. Mamadou Marouf Keita on (00223) 66722022 or Prof. Mahamadou Diakité (Permanent Secretary) at (00223) 66231191.

Appendix 2b: Verbal Household Consent form for Trial Participation

(English version, to be translated into French and Bambara and read aloud to village leaders

To be recorded electronically by a member of the LAKANA study team, on behalf of the head of the household or other person authorised to give consent for household participation

The giving and recording of consent should be confirmed by an independent witness

The persons named below affirm that they have given verbal consent and received information about the proposed trial or had this information explained to them, and that they consent to all eligible infants living in the household being enrolled in the trial. Witnesses to the consent process affirm that consent was given and information about the trial received according to the conditions laid out in the information about the trial.

Name of household

____

Name of Person giving consent for household

____

Role in household

Did the above-named person give consent? Yes/No Date

Name of Witness to Consent Procedures: ____________________________________

(*If subject is illiterate, or otherwise unable to give consent)*

____

Witness's Confirmation Yes/No Date

Name of Investigator: ____

or Authorized Representative obtaining informed consent

____

Investigator's Confirmation (initials or study code) Yes/No

Date

Appendix 3: Consent to provide study medicine to an individual infant

(English version, to be translated into French and Bambara and read aloud to village leaders

For **Parent/Guardian aged 18 years or older or married Parent/Guardian aged 16 years or older**

[If parent or guardian is younger than 18 and unmarried, or younger than 16 years of age, skip to next page]

The persons named below affirm that they have given verbal consent and received information about the proposed trial or had this information explained to them, and that they consent to the infant or infants in their care being enrolled in the trial, to receive study medicine and for information about these infants to be collected by the trial team. Witnesses to the consent process affirm that consent was given and information about the trial received according to the conditions laid out in the information about the trial.

____

Name of Household & identifier for Infant receiving study medicine

____

Name and Role of Person giving consent

Did the above-named person give consent? Yes/No

Date

Name of Witness to Consent Procedures: ____

(*If subject is illiterate, or otherwise unable to give consent)*

____

Witness's Confirmation Yes/No

Date

Name of Investigator: ____

or Authorized Representative obtaining informed consent

____

Investigator's Confirmation (initials or study code) Yes/No

Date

Appendix 3: Consent to provide study medicine to an individual infant
(English version, to be translated into French and Bambara and read aloud to village leaders

Informed Consent Form for **Parents/Guardian of participants younger than 16 years old, or unmarried participants younger than 18 years old**

The persons named below affirm that they have given verbal consent and received information about the proposed trial or had this information explained to them, and that they consent to the infant or infants in their care being enrolled in the trial, to receive study medicine and for information about these infants to be collected by the trial team. Witnesses to the consent process affirm that consent was given and information about the trial received according to the conditions laid out in the information about the trial.

____

Name of Household & identifier for Infant receiving study medicine

____

Name and Role of Person giving consent

Did the above-named person give consent? Yes/No
Date

Name of Witness to Consent Procedures: ______________________________
(*If subject is illiterate, or otherwise unable to give consent)*

____

Witness's Confirmation Yes/No
Date

Name of Investigator: ______________________________
or Authorized Representative obtaining informed consent

____

Investigator's Confirmation (initials or study code) Yes/No
Date

Appendix 4a: Information on secondary outcome data collection

(English version, to be translated into French and Bambara and read aloud to village leaders

CENTRE POUR LE DEVELOPPEMENT DES VACCINS, MALI

Director General: Samba Ousmane SOW, M.D., M.Sc., FASTMH

Professor of Medicine

Infectious Diseases Project: Measles Vaccine Initiative ♦ Enteric Disease Project ♦ Pneumococcus, Meningococcus, & *Haemophilus influenzae* Prevention Program
Malaria Project: Bandiagara Malaria Project ♦ Malaria Training Program

CVD-MALI

Large-scale Assessment of the Key health-promoting Activities of New mass drug administration regimens with Azithromycin (LAKANA)

Information about Participation in the Anti-Microbial Resistance and other sub-studies and Informed Consent Forms for individuals

This document contains information for participants in a cluster-randomised, double-blinded, parallel group, controlled trial, to test the effects of mass drug administration of azithromycin on mortality and other outcomes among 1-11-month-old infants in rural Mali. It also contains the necessary consent forms for participants to enrol in the trial.

Key Investigators

Centre pour le Développement des Vaccins–Bamako, Mali

Per Ashorn, MD, PhD [1], Ulla Ashorn, PhD [1], Yin Bun Cheung, PhD [1,2], Camilla Ducker, MSc., MBBS [3], Nigel Klein, MBBS, PhD [4], Samba O. Sow MD., MSc., FASTMH [5].

Participating Academic Institutions

[1] Tampere University, Finland; [2] Duke-NUS Medical School, Singapore; [3] Tro Da Ltd, UK; [4] University College London, UK; [5] CVD-Mali, Bamako, Mali.

Protocol version 4.0: 27. June 2022

Introduction

Thank you for participation in the LAKANA trial, which will try to determine if a drug called azithromycin can help Malian infants to enjoy better health and better rates of survival.

You will have already received information about the trial and have given consent for the trial team to ask questions about the health of the child/children in your care and for us to give them trial medicine (azithromycin or placebo).

If you would like more information about the trial or be reminded of its key aims, please speak to the members of the trial team or the *relais communautaire* who visit your village or the study team.

LAKANA sub-studies

The main LAKANA study will be implemented in approximately 1150 villages or town areas in Kayes. In some of the villages, there will be additional sub-studies, in which we will collect further information on the effects and feasibility of azithromycin distribution. Your village and others around the CSComs of Bendougouba, Dafela, Djidian, Kofeba and Koulikoro have been selected for the sub-studies. Therefore, we would like to describe them to you now and ask if you are in principle willing to participate or allow your child to participate in some of them.

In total, there will be four sub-studies that will be completed over the next 2-3 years: one on infant growth, second on the possible development of antimicrobial resistance, third on the mechnisms how azithromycin works and fourth on the feasibility of distributing azithromycin to populations in Mali. No child will be invited to participate in these sub-studies more than once or twice. Later, when we are about to do any of these sub-studies, we will ask your permission again.

Infant growth sub-study

In some earlier studies, antibiotic treatment has improved child growth. Therefore, we would also like to study if infants who receive azithromycin are bigger than infants who receive placebo.

To measure growth, we will at certain home visits invite all 6-8 month-old and 12-14 month old children to a nearby health centre or central location in village. At the health centre/central location, we will measure the child's weight, length, and arm. Growth measurement is harmless, although some infants cry a bit when their length is taken. This visit will take approximately 30 minutes plus your travel time to the location.

The Antimicrobial resistance (AMR) sub-study

When antibiotics are used, bacteria sometimes become resistant to them, leading to reduced options for treatment of children or adults with infections. In order to judge if this happens with azithromycin in Kayes, the trial team will conduct an antimicrobial

resistance (AMR) sub-study in your village.

To measure AMR, the study team will at selected home visits collect swab samples from children who are 4-14 month-old or 49-59 month-old. The samples will be collected with a thin cotton swab from the nose and rectum of the child, at your home or a central location of your village. The procedure is harmless and takes only some minutes, although swabbing the nose may sometimes feel a bit uncomfortable and some children may cry a bit. Any irritation is, however, mild and transient.

The AMR samples will be collected on four of the home visits that the LAKANA team will make: when the household joins the study and at 12, 24, and 36 months thereafter. Since the samples are taken only from children who are at the right age-bracket at these visits, the same child can provide samples only once and we will ask for your permission each time before taking any samples.

The collected samples will be stored and analysed at CVD-Mali laboratory in Bamako. Selected samples will be shipped for further analyses in collaborating centres in UK, Finland and possibly elsewhere. All samples will be coded and stored anonymously.

How azithromycin works

In order to better understand how azithromycin works in infants, the study team will carry out two sets of data collections.

At the fourth study visit, before provision of the study medicine, the study team will take a small blood sample with a finger-prick or heel-prick from all 4-11 month-old infants, at a central location of your village. The team will also collect a stool sample from the same infant. Two weeks later, you will be asked to bring the infant to a nearby health facility, for collecting another finger-prick or heel-prick blood sample and a stool sample. At this second visits, there will also be an interview about the health of the infant after she received the study medicine.

Later, at selected home visits, we will invite 6-8 month-old and 12-14 month-old infants to be brought to a health centre or another central location in the village. At the health centre or central location, a study nurse will collect a small blood sample (less than a tea-spoon) from the child's arm. Before the visit, you will be given a disposable diaper and a small plastic container. We will ask you to place the disposable diaper on the child on the morning of the health centre visit and the study team will collect a urine sample from it. In the container, we will ask you to collect a sample of the child's stool and bring it to the health centre. There will also be an interview about the health of the infant after she received the study medicine.

The health facility/central location visits will take approximately 45 minutes plus your travel time. The procedures during these visits are harmless, but blood sampling can cause some discomfort and the child may have mild tenderness and bruising following the blood being drawn. Very rarely children can feel lightheaded for a short period of time.

Since the samples are taken only from children who are at the right age-bracket at selected visits, the same child can provide samples only once or at maximum twice and we will ask for your permission each time before taking any samples.

The collected samples will be stored and analysed at CVD-Mali laboratory in Bamako.

Selected samples will be shipped for further analyses in collaborating centres in UK, Finland and possibly elsewhere. All samples will be coded and stored anonymously.

Acceptability and feasibility of azithromycin distribution

The study will also try to answer questions about the acceptability and feasibility of treating infant with azithromycin. In order to do so, the study team will ask questions about these themes during selected home visits. Questions will be about your infant's health, about the local health system and about the acceptability of azithromycin to your community, for example. Your answers will help to shape the outcomes of the study.

These additional questions will be asked at your home, by specifically trained study personnel. In total, there will be three interviews and each of them will take approximately 30 minutes. Your answers will be entirely confidential and will not be used to identify you or your family in any way.

Is participation voluntary?

Your infant's participation in these sub-studies is entirely voluntary. You do not have to take part in these, and you are free to leave them at any time without giving a reason.

What are the possible benefits of being in these sub-studies?

There may be no direct benefits to you or your infant as a result of participating in these sub-studies. However, the information obtained in these sub-studies will be important in deciding if azithromycin could improve the health of infants in Mali and other similar countries.

What kind of feedback can I expect from research results?

We will know the results of the sub-studies when the trial has finished. The study team will hold meetings to inform the community about the results.

What are the alternatives to participating in these sub-studies?

These sub-studies are an addition to the main azithromycin trial and are not a routine part of the trial's drug treatment. They are not either a part of routine health care. The alternative to participating in these sub-studies is choosing not to take part. Not participating in this sub study does not affect your right to participate in the main Azithromycin trial.

How will information and biological samples be kept private?

To protect your privacy, we will keep the information collected by the sub-studies about your infant secure. We will only allow authorized people to see it, including the study team from Mali, UK, and Finland, their representatives and the Ethics Committee in Mali, who act to protect people who take part in research. If study results are released to the public, the identities of participants will not be shared.

Biological samples taken from your child will also be stored securely at the CVD laboratory in Mali. All samples will be coded and stored anonymously.

Who can answer questions about these sub-studies?

If you have any questions regarding your infant's participation or if your infant has a problem as a result of participating in this study, you may contact at any time Dr Fatoumata Diallo on (00223) 74 60 18 19, Dr Adama Coulibaly on (00223) 66 05 04 16, Dr Fadima Cheick Haidara on (0023) 66 73 34 91, Prof Samba Sow on (00223) 76348947, or CVD-Mali, CNAM, Ex-Institut Marchoux on (00223) 20 23 60 31.

To learn more about the ethical approval of this study or your rights as a research subject, you can contact the Ethics Committee of FMPOS at (00223) 2022 5277, Prof. Mamadou Marouf Keita on (00223) 66722022 or Prof. Mahamadou Diakité (Permanent Secretary) at (00223) 66231191.

Appendix 4b: Consent form for Participation in LAKANA Sub-studies

(English version, to be translated into French and Bambara and read aloud to village leaders

For **Parent/Guardian aged 18 years or older or married Parent/Guardian aged 16 years or older**

[If parent or guardian is younger than 18 and unmarried, or younger than 16 years of age, skip to next page]

The persons named below affirm that they have given written consent and received information about the proposed LAKANA sub-studies or had this information explained to them, and that they consent to all eligible infants in their care being enrolled in the sub-studies. This includes consenting to the trial team taking all necessary biological samples. Witnesses to the consent process affirm that consent was given and information about the sub-studies received according to the conditions laid out in the trial information.

I am consenting to:

1. Participation in the growth sub study ☐
2. Participation in the antimicrobial resistance sub study ☐
3. Participation in the mechanisms sub study ☐
4. Participation in the acceptability and feasibility sub study ☐

____

Printed name of participant

____

Printed name of participant's parent/guardian

Signature or Fingerprint of Participant's Parent/Guardian ____________ Date

Name of Witness to Consent Procedures: ______________________________

(*If subject is illiterate, or unable to sign)*

____

Witness's Signature Date

Name of Investigator: ____

or Authorized Representative obtaining informed consent

____

Investigator's Confirmation (initials or study code) Yes/No
Date

Appendix 4b contd: Consent form for Participation in LAKANA Sub-studies
(English version, to be translated into French and Bambara and read aloud to village leaders

Informed Consent Form for **Parents/Guardian of participants younger than 16 years old, or unmarried participants younger than 18 years old**

The persons named below affirm that they have given written consent and received information about the proposed LAKANA sub-studies or had this information explained to them, and that they consent to all eligible infants in their care being enrolled in the sub-studies. This includes consenting to the trial team taking all necessary biological samples. Witnesses to the consent process affirm that consent was given and information about the sub-studies received according to the conditions laid out in the trial information.

I am consenting to:

1. Participation in the growth sub study ☐
2. Participation in the antimicrobial resistance sub study ☐
3. Participation in the mechanisms sub study ☐
4. Participation in the acceptability and feasibility sub study ☐

____

Printed name of participant

____

Printed name of participant's parent/guardian

____

Signature or Fingerprint of Participant's Parent/Guardian Date

Name of Witness to Consent Procedures: ______________________________

(*If subject is illiterate, or unable to sign)*

____

Witness’s Signature Date

Name of Investigator: ____

or Authorized Representative obtaining informed consent

____

Investigator’s Confirmation (initials or study code) Yes/No
Date
