# Supplementary material for: Testing the effects of mass drug administration of azithromycin on mortality and other outcomes among 1–11-month-old infants in Mali (LAKANA): study protocol for a cluster-randomized, placebo-controlled, double-blinded, parallel-group, three-arm clinical trial
Source: Trials. 2023 Jan 3;24:5. doi: 10.1186/s13063-022-06966-7 (PMC9809521; doi:10.1186/s13063-022-06966-7)
Supplement: Supplementary file 2 — Additional file 2. World Health Organization Trial Registration Data Set. [file 13063_2022_6966_MOESM2_ESM.docx]

**Supplemental Table 1: World Health Organization Trial Registration Data Set.**

| **Item** | **Information** |
| --- | --- |
| Primary registry and trial identifying number | ClinicalTrials.gov ID: NCT04424511 |
| Date of registration in primary registry | June 11, 2020 |
| Secondary identifying numbers | INV-003354 (Grantor or Funding Number: Bill and Melinda Gates Foundation) |
| Source(s) of monetary or material support | Bill and Melinda Gates Foundation |
| Primary sponsor | Tampere University |
| Secondary sponsor (s) |  |
| Contact for public queries | Per Ashorn, MD, PhD (Principal investigator, Tampere University) [per.ashorn@tuni.fi](mailto:per.ashorn@tuni.fi)  Ulla Ashorn, PhD (Co-Principal investigator, Tampere University) [ulla.ashorn@tuni.fi](mailto:ulla.ashorn@tuni.fi) |
| Contact for scientific queries | Per Ashorn, MD, PhD (Principal investigator, Tampere University) [per.ashorn@tuni.fi](mailto:per.ashorn@tuni.fi)  Ulla Ashorn, PhD (Co-Principal investigator, Tampere University) [ulla.ashorn@tuni.fi](mailto:ulla.ashorn@tuni.fi) |
| Public title | Testing the Effects of Mass Drug Administration of Azithromycin on Mortality and Other Outcomes Among 1–11-Month-Old Infants in Mali (LAKANA) |
| Scientific title | LAKANA, a Cluster-randomized, Double-blinded, Parallel Group, Controlled Trial, Testing the Effects of Mass Drug Administration of Azithromycin on Mortality and Other Outcomes Among 1–11-Month-Old Infants in Rural Mali. |
| Countries of recruitment | Mali |
| Health condition(s) or problem(s) studied | Mortality |
| Intervention(s) | Placebo Comparator: Placebo mixture  Active comparator: Azithromycin-biannually (2-dose AZI)  Active comparator: Azithromycin-quarterly (4-dose AZI) |
| Key inclusion and exclusion criteria | Inclusion Criteria:  On a cluster (village) level:   - Location within Kayes, Kita, or Koulikoro region of Mali - Considered accessible and safe by the local health authorities and research team - Considered non-urban by the local health authorities and research team - Permission from community leadership   On a household level (for trial enrollment):   - Location within a cluster that is included in the study - Verbal consent from a head of household or an authorized representative   On a child level (for receiving study medication):   - Residence in a household enrolled in the trial - Age between 29 and 364 days - Verbal consent from at least one caregiver   Exclusion Criteria:  On child level (for not receiving study medication):   - Weight below 3.0 kg - Known allergy to macrolides |
| Study type | Type of study: interventional  Method of allocation: cluster randomized in a ratio of 3:2:4 to three arms  Masking: quadruple (Participant, Care provider, Investigator, Outcome’s assessor) |
| Date of first enrolment | October 15, 2020 |
| Target sample size | 1150 clusters recruited, or 35,650 infants treated at Mass Drug Administration #1, whichever is achieved first. |
| Recruitment status | Recruiting |
| Primary outcome(s) | Mortality (deaths per 1,000 years at risk among children who were 1-11-month-old at MDA) |
| Key secondary outcomes | Prevalence of antimicrobial resistance towards macrolides and other antibiotics  Episodes of acute respiratory infection, malaria, and diarrhea  Infant and young child growth  Malaria parasitemia, inflammation  Feasibility of azithromycin MDA implementation including economic analysis  Incidence of serious adverse events (SAE) and adverse events (AE)  Mortality among children, who were 12–59-month-old at the MDA |
